# Specific Learning Disorders: Variation Analysis of 15 Candidate Genes in 9 Multiplex Families

**DOI:** 10.3390/medicina59081503

**Published:** 2023-08-21

**Authors:** Francesco Calì, Francesco Domenico Di Blasi, Emanuela Avola, Mirella Vinci, Antonino Musumeci, Angelo Gloria, Donatella Greco, Daniela Rita Raciti, Alessandro Zagami, Biagio Rizzo, Santina Città, Concetta Federico, Luigi Vetri, Salvatore Saccone, Serafino Buono

**Affiliations:** 1 Oasi Research Institute—IRCCS, Via Conte Ruggero 73, 94018 Troina, Italy; fcali@oasi.en.it (F.C.); fdiblasi@oasi.en.it (F.D.D.B.); fbuono@oasi.en.it (S.B.); 2 Department Biological, Geological and Environmental Sciences, University of Catania, Via Androne 81, 95124 Catania, Italy

**Keywords:** Specific Learning Disorder (SLD), dyslexia, next-generation sequencing, multiplex SLD families, single nucleotide polymorphisms

## Abstract

*Background and Objectives*: Specific Learning Disorder (SLD) is a complex neurobiological disorder characterized by a persistent difficult in reading (dyslexia), written expression (dysgraphia), and mathematics (dyscalculia). The hereditary and genetic component is one of the underlying causes of SLD, but the relationship between genes and the environment should be considered. Several genetic studies were performed in different populations to identify causative genes. *Materials and Methods*: Here, we show the analysis of 9 multiplex families with at least 2 individuals diagnosed with SLD per family, with a total of 37 persons, 21 of whom are young subjects with SLD, by means of Next-Generation Sequencing (NGS) to identify possible causative mutations in a panel of 15 candidate genes: *CCPG1*, *CYP19A1*, *DCDC2*, *DGKI*, *DIP2A*, *DYM*, *GCFC2*, *KIAA0319*, *MC5R*, *MRPL19*, *NEDD4L*, *PCNT*, *PRMT2*, *ROBO1*, and *S100B*. *Results:* We detected, in eight families out nine, SNP variants in the *DGKI*, *DIP2A*, *KIAA0319*, and *PCNT* genes, even if in silico analysis did not show any causative effect on this behavioral condition. In all cases, the mutation was transmitted by one of the two parents, thus excluding the case of de novo mutation. Moreover, the parent carrying the allelic variant transmitted to the children, in six out of seven families, reports language difficulties. *Conclusions*: Although the present results cannot be considered conclusive due to the limited sample size, the identification of genetic variants in the above genes can provide input for further research on the same, as well as on other genes/mutations, to better understand the genetic basis of this disorder, and from this perspective, to better understand also the neuropsychological and social aspects connected to this disorder, which affects an increasing number of young people.

## 1. Introduction

“Specific Learning Disorders” (SLDs) constitute a set of heterogeneous disorders manifested by difficulties in learning and in the use of academic skills (reading, written expression, and mathematics). Different SLDs often co-occur in the same child; therefore, the presence of dyslexia and/or dysgraphia and/or dyscalculia can also be observed. SLDs express with overall intact cognitive functioning, absence of neurological and sensory disorders, and significant and persistent limitations in school and daily life activities. They can also be associated with other neurodevelopmental disorders, such as inattentive-type “Attention Deficit Hyperactivity Disorder” (ADHD) or motor coordination disorder [1]. SLD recognition has evolved over time. In the early 20th Century, learning difficulties were often misunderstood or attributed to intellectual deficiencies. Concepts of neurodevelopmental disorders were not well established, and educational approaches were often not tailored to individual needs. Then, the concept of dyslexia gained prominence as researchers and educators began to recognize specific difficulties in reading, despite average or above-average intelligence. The term “dyslexia” was coined to describe this condition. More recently, research expanded to include dyscalculia (mathematics difficulties) and dysgraphia (writing difficulties), leading to the broader understanding of SLD [2,3]. Indeed, among the learning disabilities, dyslexia has been and is currently the most studied disorder, as the inefficient use of reading is a highly limiting condition, much more than it was fifty or a hundred years ago. Thus, in the last 50 years, scientific research has also extended its interest to other SLDs by integrating the interdisciplinary knowledge of neuroscience, psychology, genetics, and education. The increased recognition of SLDs in modern times is attributed to advancements in diagnostic criteria, greater awareness and research, improved educational practices, better understanding of neurodevelopment, screening programs, changing educational demands, environmental factors, and broader diagnostic criteria. The greater knowledge on SLDs has consequently increased information, training, and sensitivity towards this phenomenon in society, in health systems, in school systems, and in families. The demand for diagnosis and care that people with SLD have, therefore, increased significantly. After all, the problem does not only concern the assessment of the disorder, but also the treatment. It should be emphasized that early diagnosis can improve the prognosis, reducing the risk of chronicity and helping to obtain positive effects not only for the child and his family, but also for society as a whole, with a containment of costs for assistance [2,4].

The broad spectrum of the disorder, the different orthographic characteristics of languages [5], and the different criteria and tests adopted for the diagnosis influence SLD prevalence estimation in different countries. According to *Diagnostic and Statistical Manual of Mental Disorders-5* (DSM-5), the prevalence range of SLD is estimated to be about 5% to 15% worldwide [6]. In Italy, the prevalence of SLD among students is between 2.5% and 3.5% (National Institutes of Health). However, this figure is very uneven among the various Italian regions, and above all, in the southern regions, including Sicily, where the phenomenon appears to be underestimated [7], with approximately 250.000 students presenting with a specific learning difficulty [8].

Studies carried out in recent years on dyslexic families and twins largely confirm the genetic predisposition to SLD [9], even if the genetics of SLD is currently at an early stage of knowledge. Thus, while genetics play a substantial role, twin studies also highlight the importance of environmental factors, which may include unique educational experiences, early interventions, and individualized learning environments [10,11,12,13].

The genetic architecture of SLD is complex and involves a combination of genetic factors that contributes to an individual’s susceptibility to these disorders. Some key aspects concern its polygenic nature; the presence of common and rare variants, often converging on specific biological pathways and networks related to brain development; neurotransmitter function; and neuronal communication [14,15,16,17,18]. Some chromosomal regions were originally associated to SLD, such as 3p12, 6p22, and 15q21 [19,20,21,22,23,24], and over the past decade, several candidate genes have been identified that may contribute to susceptibility to SLD [25], some of which have been implicated in specific biological processes, such as neuron migration during brain development (e.g., *DCDC2*, *DYX1C1*, *KIAA0319*, and *ROBO1*) [25,26,27]. They are thought to be involved in the regulation of neuronal migration and dendrite and axon growth through the regulation of primary cilia formation and function. This suggests that susceptibility to SLD can be considered as the mildest expression of a pathological spectrum that affects neuronal development and connection and that, in its most severe forms, is expressed in severe brain malformations with severe intellectual disability. Mutations of both *DYX1C1* and *DCDC2* genes have been found in patients with primary ciliary dyskinesia and nephronophthisis-related ciliopathies, respectively [28,29].

All studies to date, while clearly indicating the genetic nature of SLD and while providing insights into the pathogenetic mechanism, have not yet fully clarified the cause of this disorder, or provided unambiguous results [30,31]. Certainly, today, we learn that complex neurodevelopmental disorders have a polygenic nature, where genetic and many other factors contribute simultaneously to environmental contexts to influence a phenotype. In a Finnish study, it was specifically observed how the shared reading experience between parent and child has a positive effect on both oral language development and the development of literacy skills in general [32].

The recent progress on gene sequencing technologies, such as Next-Generation Sequencing (NGS), has provided the scientific community a methodology for genetic analysis in diseases with very similar or overlapping phenotypes. Kovas and Plomin [33] conducted a study on twins, and the results showed a substantial genetic influence on individual differences in learning skills, such as reading and calculation. Multivariate genetic research has also shown that the same set of genes is largely responsible for the genetic influence on different cognitive areas. However, what differentiates these skills is largely the environment.

Recently, genetic association studies have been conducted via genome-wide association studies (GWASs) on dyslexia. Data were collected on a cohort of 51,800 adults who self-reported a diagnosis of dyslexia and 1,087,070 controls. The mean age of cases and controls was 49.6 years and 51.7 years, respectively, ranging from 18 to 110 years, with a higher prevalence of dyslexia in younger participants (5.34% in those aged 20 to 30) compared to older participants (3.23% in those aged 80 to 90). The negative linear relationship between dyslexia prevalence and participant age was expected, given that screening for specific learning disabilities has only become common in recent decades [30]. The study results identified 173 significantly associated genes within the set of credible variants, also noting that missense variants were more common (by 55%) than coding variants. Of the 173 significant genes, 129 could be functionally annotated. Among them, genetic correlations were estimated for 98 different phenotypic traits, taking into account brain subcortical structure volumes, total cortical surface area, and relative thickness. Next, the Enhancing Neuro Imaging Genetics through Meta-Analysis (ENIGMA) consortium estimated a total of 63 traits with significant genetic correlates with dyslexia. The ENIGMA Consortium in a study has identified a positive genetic correlation between hearing difficulties and dyslexia, which is consistent with the genetic correlations reported for childhood reading ability, and which suggests that hearing problems at an early age could influence the acquisition of phonological processing skills [34]. Another example of a genetic correlation with dyslexia involves equal use of the hands, but not left-handed, supporting theories linking ambidexterity and dyslexia [35].

NGS has revolutionized diagnostic genetic testing by replacing the “gene-by-gene” approach with a gene panel strategy. This new approach is particularly promising for the diagnosis of diseases that are characterized by strong clinical and genetic heterogeneity or for complex diseases, such as SLD. Although there is to date no clear evidence for the etiology of SLD, several susceptibility genes have been identified [36,37], and here we show data on 15 genes related to SLD, by using a previously described NGS-based procedure [38], in 21 subjects belonging to 9 unrelated families, with at least 1 child with SLD for each family. The selected genes were (in alphabetical order): (1) *Cell cycle progression 1* (*CCPG1*); (2) *Cytochrome P450 family 19 subfamily A member 1* (*CYP19A1*); (3) *Doublecortin domain containing 2* (*DCDC2*); (4) *Diacylglycerol kinase iota* (*DGKI*); (5) *Disco interacting protein 2 homolog A* (*DIP2A*); (6) *Dymeclin* (*DYM*); (7) *GC-rich sequence DNA-binding factor 2* (*GCFC2*); (8) *KIAA0319* (*KIAA0319*); (9) *Melanocortin 5 receptor* (*MC5R*); (10) *Mitochondrial ribosomal protein L19* (*MRPL19*); (11) *Neural precursor cell expressed developmentally down-regulated protein 4* (*NEDD4L*); (12) *Pericentrin* (*PCNT*); (13) *Protein arginine methyltransferase 2* (*PRMT2*); (14) *Roundabout guidance receptor 1* (*ROBO1*); (15) *S100 calcium binding protein B* (*S100B*). A brief description of the product function, together with the main genomic properties and the relative references, is detailed in Table 1.

The aim of this study was to obtain information on the genetic bases of SLD by analyzing families in which there are subjects with this characteristic, by means of mutational analysis of a large number of genes using NGS. We selected the genes shown in Table 1, as they were, at the time of the beginning of the study, excellent candidate genes, in some of which we have in fact detected nucleotide variants segregated in the analyzed families.

## 2. Materials and Methods

### 2.1. SLD Diagnosis of the Subject

The study was performed on 21 siblings with SLD (12 males, 9 females; mean age 13.4 ± 3.6 year; with a clinical diagnosis of SLD). The subjects, all Caucasian and of Sicilian ancestry, were recruited from the diagnostic department of Oasi Research Institute, in Troina (Italy). In Italy, the diagnosis of SLD is based on the indications of the Consensus Conference [8], which, in turn, partly derive from the ICD-10 [49] and DMS-5 [6] guidelines. According to the above-mentioned document, the learning disorder criterion is based on the reading, and/or writing, and/or math performance below the mean for the same age and/or school degree (cut-offs: z-score < 2 standard deviation from the mean in speed scores, a score < 5th percentile in the accuracy scores).

A protocol of standardized tests—including a cognitive and neuropsychological test, behavioral and adaptive checklist, academic skills test, language and motor test—were carried out. Furthermore, extensive clinical and instrumental investigations—which included neurological, ophthalmological, and orthoptic examinations; hearing tests; and EEG—were performed. The assessment of a possible presence of the disorder in the parents was confirmed during the history taking.

### 2.2. Genomic DNA Preparation and Mutational Analysis

Genomic DNA was isolated from peripheral blood leucocytes, according to standard protocols. Mutation analysis of 15 genes (all coding exons and splice sites) was carried out using a NGS panel with the following genes: *CCPG1* (previously called *DYX1C1*), *CYP19A1*, *DCDC2*, *DGKI*, *DIP2A*, *DYM*, *GCFC2* (previously called *C2Orf3*), *KIAA0319*, *MC5R*, *MRPL19*, *NEDD4L*, *PCNT*, *PRMT2*, *ROBO1*, *S100B* (Table 1). Analysis was performed using “Ion AmpliSeq™ Designer” (Life Technologies, Carlsbad, CA, USA, IAD79247). A total of 422 amplicons were analyzed in two different pools. The overall coverage of all genes was 98%. The library was generated using 10 ng of genomic DNA and the Ion AmpliSeq™ Library Kit for Chef DL8. A dilution of the library was used for clonal amplification using an ION-CHEF instrument. The amplification product was loaded onto a 530 IonChip and then sequenced according to the “Ion S5 Sequencing Kit” protocol. All detected mutations were confirmed by conventional Sanger sequencing and verified in all the family members.

### 2.3. Bioinformatic Analysis

The study was conducted between 2016 and 2020. The identified variants were filtered according to recessive/de novo pattern of inheritance, gene features, and MAF < 1%, using as references 1000 Genomes, ESP6500, ExAC, and gnomAD. According to the 28 criteria in the “American College of Medical Genetics” (ACMG) guidelines, variants are classified into five tiers: Pathogenic (P), Likely pathogenic (LP), Uncertain significance (VUS), Likely benign (LB), and Benign (B), depending on the applied criteria. The VarSome germline variant [50] classifier automatically generates a pathogenicity recommendation based on these ACMG guidelines.

## 3. Results

One patient with SLD from each family was analyzed using an NGS panel with 15 candidate genes (Table 1).

**Figure 1:**
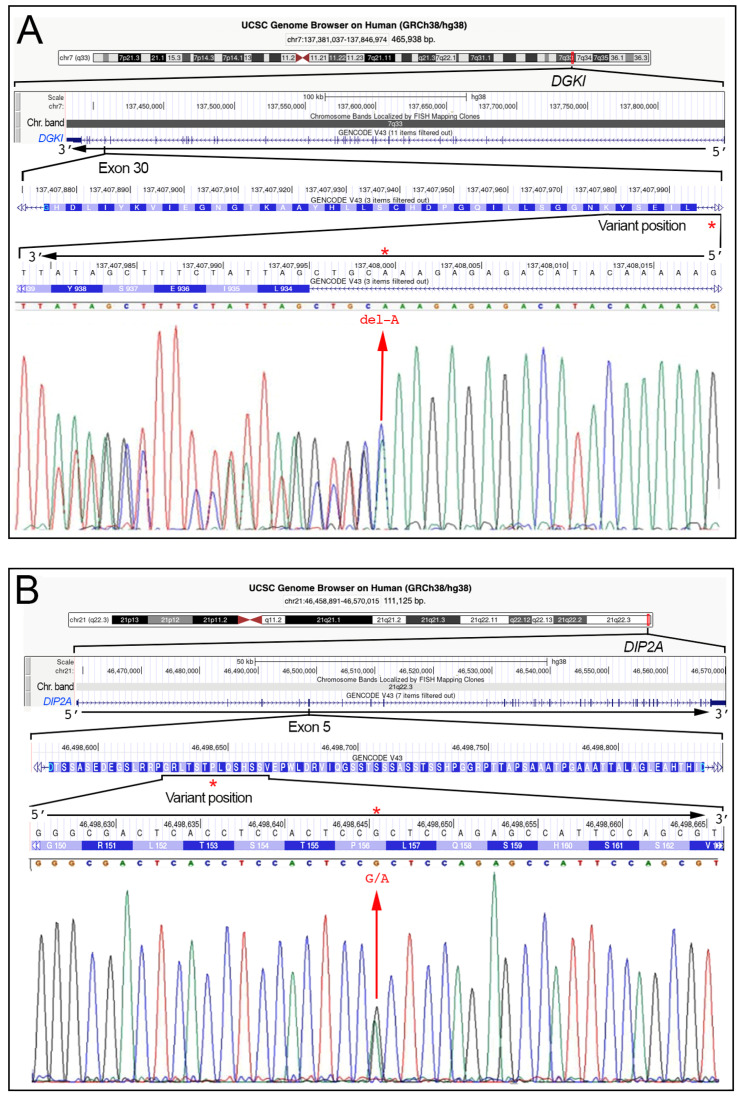

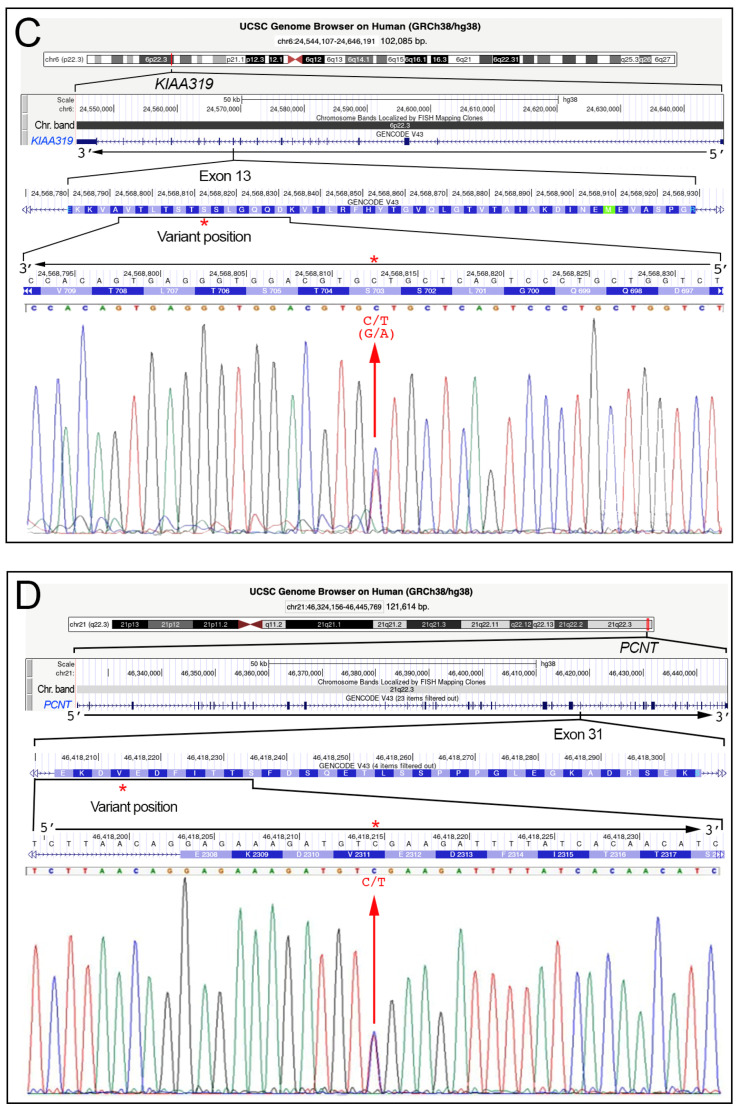
Genomic features of the four genes containing the nucleotide variations detected in the SLD families. (**A**–**D**) show data on *DGKI*, *DIP2A*, *KIAA319*, and *PCNT* genes, respectively. Each panel shows, from upper to bottom, (1) the ideogram of the chromosome (chr6, chr7, chr21) with the location of the gene (red rectangle), and the corresponding chromosomal band (Chr. band), (2) the exon/intron organization of the gene, (3) the aminoacidic sequence of the involved exon, (4) the enlargement of the gene region containing the variant, and (5) the relative electropherogram obtained with the Sanger method. The c.2824-4del (*DGKI* gene), c.468G>A (*DIP2A* gene), c.2108G>A (*KIAA0319* gene), and c.6933C>T (*PCNT* gene) variants are highlighted by a red arrow and by a red asterisk. Images corresponding to the above (1) to (4) points were obtained from the University of California Santa Cruz (UCSC) genome browser (http://genome.ucsc.edu, accessed 11 July 2023).

Variations in four genes (*DGKI*, *DIP2A*, *KIAA0319*, and *PCNT*) were detected in the analyzed patients. One of these is a missense mutation (c.1218G>A in the *KIAA0319* gene), another two are synonymous substitution (c.468G>A in the *DIP2A* gene and c.6933C>T in the *PCNT* gene), and the fourth is a 4-bp deletion (c.2824-4del in the *DGKI* gene). These nucleotide variations were confirmed by Sanger sequencing (Figure 1), and the identified variants, by in silico analysis, filtered according to MAF <1% were classified as benign or likely benign, according to the “American College of Medical Genetics” criteria (Table 2).

All identified mutations were reanalyzed in all family members, and the variants have been identified in eight out of nine of the studied families. No variants in the analyzed 15 genes were found in the ninth family. Each subject was described considering the typical phenotype (dyslexia, dysgraphia, dyscalculia), age, gender, and molecular phenotype. Moreover, all family members were assessed in the anamnestic phase through a direct interview, and the presence of one or both parents endowed with Learning Difficulties (LDs) encountered at school has been highlighted (Table 3). The identified variants were detected in heterozygosity in at least two components of each of the nine families here studied. The allelic variants have been transmitted, in six cases out of seven, from a parent with learning disabilities, even if not all SLD children have inherited the mutated allele. It should be stressed that considering the 7 families where a mutated allele was detected, and the parents were present, all carrier parents, except 1, accounted for LD, and 12 offspring with SLD out of 21 were carriers of a mutated allele (Table 3).

## 4. Discussion

Our study, performed on SLD subjects belonging to the Italian population, is one of the few centered on the mutational analysis of several candidate genes. Among the 15 analyzed genes, we found allelic variants in 4 of these genes, namely, *DGKI*, *DIP2A*, *KIAA0319*, and *PCNT*. These variants were detected in eight different families out of nine (Table 3), where the transmitter parent was in six cases out of seven endowed with a learning difficulty.

According to the current literature, rare variants have been reported in few studies, significantly associated with SLD; a translocation breakpoint at 15q21 in the *CCPG1* (previously called *DYX1C1*) gene was reported as the first gene to be implicated in dyslexia [51], further to other large deletions/insertions at chromosome 15 that were found [52]. A rare variant was reported at the *ATPase secretory pathway Ca2+ transporting 2* (*ATP2C2*) gene [53] or *sprouty RTK signaling antagonist 1* (*SPRY1*) gene [54], and a 452.4 Kb de novo heterozygous micro-deletion in chromosomal region 1p34.3 in a patient with dyslexia and attention-deficit/hyperactivity disorder was reported [55]. Several studies, indeed, have tried to investigate the possible cause of SLD; however, no agreement regarding the exact causes and nature of SLD has so far been found among the scientific communities [25]. Comorbidity, of course, makes differential diagnosis an even more complicated task [56]. A recent study discovering 42 further new different loci associated with dyslexia demonstrated the difficulty in the study of SLD [30], typically observed as a heterogeneous condition.

Our study, performed on SLD subjects belonging to the Italian population, is one of the few centered on the mutational analysis of several candidate genes currently related to SLD. Some of these subjects are children from the same parents, thus allowing us to highlight the high level of heredity of the detected variants. Two of these variants (*DIP2A*: c.468G>A and *KIAA0319*: c.2108G>A) were segregated in one family, one (*PCNT*: c.6933C>T) was segregated in two different families, and the fourth (*DGKI*, c.2824-4del) was segregated in five different families. Except in one family (where the parents were not present), in the other eight, the mutation was transmitted by one of the two parents, thus excluding the case of de novo mutation.

The in silico analysis of the four variants, using the criteria of the “American College of Medical Genetics”, did not provide any causative effects of the SLD, the results being obtained as “likely benign” in three out four cases and “benign” in one case. Except for the variant c.2824-4del (*f* = 0.0), all others are rare (*f* value between 0.00028 and 0.005), and there is no large difference in frequency among various other populations (data not shown). We think that for a significant relationship, considering this specific behavioral disorder, which cannot be considered as a clear pathological condition, we have to integrate these results with the following observations: (1) the in silico “Likely Benign” analysis did not denote any pathogenic variants (Table 2); (2) the variants were inherited from parents with learning disabilities (see Table 3), that even if they cannot be considered SLD, could be an indication of a problematic behavioral condition; (3) each identified variant was segregated in more than 50% of affected members of the SLD subjects (Table 3); (4) in almost all families, there are children and at least a parent carrying the variants. Thus, for a specific causative effect of the variant here detected, we think other data should be necessary, possibly by expansion of the sample size. Moreover, it should be stressed that the mutations in exons detected in the present analysis, which was focused on exonic sequences and the adjacent splice sites, as well as the possible presence of mutations in the introns, can also determine long-range effects not only in the expression of the gene carrying the mutation, but also in contiguous genes, as observed, for example, for the SNP rs12913832 located in exon 86 of the *HERC2* gene determining the alteration of the expression level of the adjacent *OCA2* gene [57,58], a gene involved in human pigmentation.

The available studies performed on SLD have identified many genes/regions; however, the mutation analysis of these does not seem to be efficient in identifying pathogenic variants and disease genes. But, for a correct analysis of the results, we have to stress, for example, that the marked clinical and genetic heterogeneity of the disorder or the environmental exposure to unknown factors, which can result in different phenotypic outcomes, even with the same genotype, should be considered. To date, therefore, the genetic basis of the SLD disorder has not yet been well defined, and despite several pieces of evidence that SLD is highly heritable, its exact biological basis remains elusive.

Genomic analysis by means of NGS, as we obtained in this work, confirms the multifactorial nature of SLD, and identifying new gene variants associated with it contributes to better characterizing the molecular and neurobiological mechanisms related to the specific learning problems, namely, dyslexia, dysgraphia, and dyscalculia.

## 5. Conclusions

The current ability to generate rapidly and at an affordable cost the sequencing of the entire exome, and even the genome, could lead to greater advantages. Prospects in the genetic study of SLD might include analysis of related specific phenotypes (dyslexia, dysgraphia, and dyscalculia). The combination of such approaches in a greater sample is likely to lead to significant discoveries. Working on this perspective can allow us in the future to better understand also the neuropsychological and social aspects connected to this disorder, which affects an increasing number of young people, and finally, understanding the molecular and neurobiological mechanisms of SLD could teach us something about general cognition, brain development processes, and the specific evolution of the human brain. Since SLD is a multigenic trait, the contribution of each variant could also be relevant as a factor of the vulnerability towards certain environmental stimuli. Our work has highlighted, in the families of the Caucasian population analyzed, some polymorphisms connected to this condition, even if these data certainly cannot be considered definitive, since it is necessary to collect further and more numerous observations. But, with the panel of genes that we have described and implemented with non-coding sequences, it will be possible in the future to better define the contribution of each of these genes to SLD.

## Figures and Tables

**Table 1:** Genomic features of the 15 genes used in the NGS panel.

Gene	Gene Product Function	Refs. ^(a)^	Position ^(b)^ (chr Band)	Genomic Size ^(b)^ (Kb)	RefSeq. Transcript ^(c)^
*CCPG1*	Related to the cell cycle regulation and cell division processes.	[39]	15q21.3	53.121	NR_037923.1
*CYP19A1*	Product is an enzyme involved in the steroid hormone conversion.	[40,41,42]	15q21.2	130.540	NM_000103.4
*DCDC2*	Involved in the formation of neuronal circuits, and neuronal migration.	[9,26]	6p22.3	186.305	NM_016356.5
*DGKI*	Involved in cellular signaling pathway by diacylglycerol phosphorylation, such as cell proliferation and differentiation, synaptic plasticity, neuronal signaling.	[43]	7q33	465.938	NM_001321708.2
*DIP2A*	Involved in various cell processes, such as proliferation, differentiation processes; Implicated in neurogenesis and neuronal differentiation.	[41,44]	21q22.3	111.125	NM_015151.4
*DYM*	Involved in various cellular processes related to the cellular homeostasis.	[45]	18q21.1	419.329	NM_001353214.3
*GCFC2*	Gene regulation and maintaining genome stability.	[41,46]	2p12	48.211	NM_003203.5
*KIAA0319*	Plays a role in brain development related to neuronal migration and neural connectivity.	[9,26,41]	6p22.3	102.085	NM_014809.4
*MC5R*	Transmembrane protein involved in various cell processes, such as skin pigmentation, immunomodulation, thermoregulation.	[45]	18p11.21	3.175	NM_005913.3
*MRPL19*	Mitochondrial protein involved in mitochondrial function and cellular metabolism	[41]	2p12	23.951	NM_014763.4
*NEDD4L*	Protein involved in ubiquitination of various proteins, regulating protein levels and functions within cells.	[45,47,48]	18q21.31	357.314	NM_001144967.3
*PCNT*	A component of the centrosome and involved in various processes, such as cell division and organization of the microtubule network.	[41]	21q22.3	121.614	NM_006031.6
*PRMT2*	Methyltransferase involved in various processes, such as gene expression, RNA processing, cell signaling.	[41]	21q22.3	29.451	NM_206962.4
*ROBO1*	Cell surface receptor involved in axon guidance during neural development.	[9,26,41]	3p12.3	1170.760	NM_002941.4
*S100B*	Calcium-binding protein involved in various cell processes, such as neurological function, immune response, cell cycle regulation.	[41]	21q22.3	6.479	NM_006272.3

^(a)^ References indicating the involvement of the gene in SLD in the period when the research was conducted. ^(b)^ From UCSC Genome Browser (GRCh38/hg38): http://genome.ucsc.edu. ^(c)^ From NCBI Resources: www.ncbi.nlm.nih.gov. Accessed on 8 July 2023.

**Table 2:** In silico analysis of the four variants identified.

Gene	Ref. seq.	DNA Variant	Protein Variant	SNP-ID ^(a)^	VarSome (ACMG) ^(b)^	GnomAD Exomes ^(c)^	gnomAD Genomes ^(c)^	TSI 1000G ^(d)^	Clinical Variant
*DGKI*	NM_004717.3	c.2824-4del	==	rs1184296555	L.B.	*f* = 0.0	*f* = 0.0	*f* = nf	N.D.
*DIP2A*	NM_015151.4	c.468G>A	p.(Pro156=)	rs367616491	L.B.	*f* = 0.00028	*f* = 0.00042	*f* = nf	N.D.
*KIAA0319*	NM_014809.4	c.2108G>A	p.(Ser703Asn)	rs138160539	L.B.	*f* = 0.00134	*f* = 0.00121	*f* = 0.005	L.B.
*PCNT*	NM_006031.6	c.6933C>T	p.(Val2311=)	rs148444313	Benign	*f* = 0.00372	*f* = 0.00360	*f* = 0.0	C.i.p.

^(a)^ Data from NCBI dbSNP (www.ncbi.nlm.nih.gov/snp/, accessed on 10 July 2023). ^(b)^ VarSome-implemented ACMG criteria (for interpretation of the clinical significance of sequence variants). ^(c)^ Total allele frequency. No data available in the Human Gene Mutation Database (HGMD) for all the variants. ^(d)^ TSI (Tuscans from Italy) data from 1000G (1000 Genomes Browser from Ensemble; accessed on 10 July 2023). nf = not found; L.B.: Likely Benign; N.D.: no data available; C.i.p.: Conflicting interpretations of pathogenicity.

**Table 3:** NGS analysis performed in SLD multiplex families. All the children presented with SLD.

Family	Code	Sex and Parents	Age	Phenotype	*DGKI* c.2824-4del	*DIP2A* c.468G>A p.(Pro156=)	*KIAA0319* c.2108G>A p.(Ser703Asn)	*PCNT* c.6933C>T p.(Val2311=)
F1	02008	Female	21	D, DS	Heterozygous		Heterozygous	
	04835	Female	25	D, DY				
	02008M	Mother	62	LD			Heterozygous	
	02008P	Father	62	//	Heterozygous			
F2	04735	Female	22	D, DS, DY				Heterozygous
	04735F	Male	19	D, DS, DY				Heterozygous
	04735F1	Male	15	D, DS, DY				
	04735M	Mother	48	//				
	04735P	Father	53	LD				Heterozygous
F3	04802	Male	26	D, ADHD		Heterozygous		
	04883	Male	25	D, DS, DY		Heterozygous		
F4	04833	Male	17	D, DS, DY				
	04833F	Male	17	D, DS, DY				
	04833S	Female	21	D, DS, DY				
	04833S1	Female	16	D, DS, DY	Heterozygous			
	04833M	Mother	43	//	Heterozygous			
	04833P	Father	47	LD				
F5	04966F	Male	16	D, DS, DY, ADHD				
	04966	Female	22	D, DS	Heterozygous			
	04966M	Mother	50	//				
	04966P	Father	52	LD	Heterozygous			
F6	05034	Male	16	D, DS				
	05034F	Male	16	D, DS				
	05034M	Mother	40	//				
	05034P	Father	53	//				
F7	05170	Male	22	D, DS, DY	Heterozygous			
	05170S	Female	25	D, DS, DY				
	05170M	Mother	51	LD	Heterozygous			
	05170P	Father	55	LD				
F8	05461	Male	26	D, DS, DY	Heterozygous			
	05461S	Female	21	D, DS, DY	Heterozygous			
	05461M	Mother	52	LD	Heterozygous			
	05461P	Father	55	//	Heterozygous			
F9	05640	male	18	D, DS				Heterozygous
	05640S	female	21	D, DS				Heterozygous
	05640M	Mother	44	LD				Heterozygous
	05640P	Father	50	LD				

D: Dyslexia; DS: dysgraphia; DY: dyscalculia; ADHD: Attention-Deficit/Hyperactivity Disorder; LD: learning difficulties.

## Data Availability

Not applicable.

## References

[1] Brimo K., Dinkler L., Gillberg C., Lichtenstein P., Lundström S., Åsberg Johnels J. (2021). The co-occurrence of neurodevelopmental problems in dyslexia. Dyslexia.

[2] Grigorenko E.L., Compton D.L., Fuchs L.S., Wagner R.K., Willcutt E.G., Fletcher J.M. (2020). Understanding, educating, and supporting children with specific learning disabilities: 50 years of science and practice. Am. Psychol..

[3] Shaywitz S.E., Shaywitz B.A. (2005). Dyslexia (specific reading disability). Biol. Psychiatry.

[4] Smart D., Youssef G.J., Sanson A., Prior M., Toumbourou J.W., Olsson C.A. (2017). Consequences of childhood reading difficulties and behaviour problems for educational achievement and employment in early adulthood. Br. J. Educ. Psychol..

[5] Ziegler J.C., Goswami U. (2005). Reading acquisition, developmental dyslexia, and skilled reading across languages: A psycholinguistic grain size theory. Psychol. Bull..

[6] APA—American Psychiatric Association (2013). Diagnostic and Statistical Manual of Mental Disorders.

[7] Leonardi M.M., Di Blasi F.D., Savelli E., Buono S. (2021). Reading and spelling disorders in a school-based population screening in Sicily (Italy). Dyslexia.

[8] ISS-Istituto Superiore di Sanità (2022). Consensus Conference. Disturbi Specifici dell’apprendimento. Sistema Nazionale per le Linee Guida. https://www.iss.it/documents/20126/8331678/LG-389-AIP_DSA.

[9] Erbeli F., Hart S.A., Taylor J. (2018). Longitudinal associations among reading-related skills and reading comprehension: A twin study. Child. Dev..

[10] Gayan J., Olson R.K. (2001). Genetic and environmental influences on ortho- graphic and phonological skills in children with reading disabilities. Dev. Neuropsychol..

[11] Harlaar N., Spinath F.M., Dale P.S., Plomin R. (2005). Genetic influences on early word recognition abilities and disabilities: A study of 7-year-old twins. J. Child. Psychol. Psychiatry.

[12] Friend A., DeFries J.C., Wadsworth S.J., Olson R.K. (2007). Genetic and environmental influences on word recognition and spelling deficits as a function of age. Behav. Genet..

[13] Wray N.R., Lee S.H., Mehta D., Vinkhuyzen A.A.E., Dudbridge F., Middeldorp C. (2014). Research review: Polygenic methods and their application to psychiatric traits. J. Child. Psychol. Psychiatry.

[14] Hannula-Jouppi K., Kaminen-Ahola N., Taipale M., Eklund R., Nopola-Hemmi J., Kääriäinen H., Kere J. (2005). The axon guidance receptor gene ROBO1 is a candidate gene for develop- mental dyslexia. PLoS Genet..

[15] Elbert A., Lovett M.W., Cate-Carter T., Pitch A., Kerr E.N., Barr C.L. (2011). Genetic variation in the KIAA0319 5′ region as a possible contribu- tor to dyslexia. Behav. Genet..

[16] Kidd T., Brose K., Mitchell K.J., Fetter R.D., Tessier-Lavigne M., Goodman C.S., Tear G. (1998). Roundabout controls axon cross- ing of the CNS midline and defines a novel subfamily of evolution- arily conserved guidance receptors. Cell.

[17] Poon M.W., Tsang W.H., Chan S.O., Li H.M., Ng H.K., Waye M.M.Y. (2011). Dys- lexia-associated kiaa0319-like protein interacts with axon guidance receptor nogo receptor 1. Cell. Mol. Neurobiol..

[18] Burbridge T., Wang Y., Volz A., Peschansky V., Lisann L., Galaburda A., Turco J.L., Rosen G. (2008). Postnatal analysis of the effect of embryonic knockdown and overexpression of candidate dyslexia susceptibility gene homolog Dcdc2 in the rat. Neuroscience.

[19] Tran C., Wigg K.G., Zhang K., Cate-Carter T.D., Kerr E., Field L.L., Kaplan B.J., Lovett M.W., Barr C.L. (2014). Association of the *ROBO1* gene with reading disabilities in a family-based analysis. Genes. Brain Behav..

[20] Platko J.V., Wood F.B., Pelser I., Meyer M., Gericke G.S., O’Rourke J., Birns J., Purcell S., Pauls D.L. (2008). Association of reading disability on chromosome 6p22 in the Afrikaner population. Am. J. Med. Genet. B Neuropsychiatr. Genet..

[21] Francks C., Paracchini S., Smith S.D., Richardson A.J., Scerri T.S., Cardon L.R., Marlow A.J., MacPhie I.L., Walter J., Pennington B.F. (2004). A 77-kilobase region of chromosome 6p22.2 is associated with dyslexia in families from the United Kingdom and from the United States. Am. J. Hum. Genet..

[22] Smith S.D., Kimberling W.J., Pennington B.F., Lubs H.A. (1983). Specific reading disability: Identification of an inherited form through linkage analysis. Science.

[23] Chapman N.H., Igo R.P., Thomson J.B., Matsushita M., Brkanac Z., Holzman T., Berninger V.W., Wijsman E.M., Raskind W.H. (2004). Linkage analyses of four regions previously implicated in dyslexia: Confirmation of a locus on chromosome 15q. Am. J. Med Genet..

[24] Grigorenko E.L., Wood F.B., Meyer M.S., Hart L.A., Speed W.C., Shuster A., Pauls D. (1997). Susceptibility loci for dis- tinct components of developmental dyslexia on chromosomes 6 and 15. Am. J. Hum. Genet..

[25] Erbeli F., Rice M., Paracchini S. (2022). Insights into Dyslexia Genetics Research from the Last Two Decades. Brain Sci..

[26] Diaz R., Kronenberg N.M., Martinelli A., Liehm P., Riches A.C., Gather M.C., Paracchini S. (2022). KIAA0319 influences cilia length, cell migration and mechanical cell–substrate interaction. Sci. Rep..

[27] Paniagua S., Cakir B., Hu Y., Kiral F.R., Tanaka Y., Xiang Y., Patterson B., Gruen J.R., Park I.-H. (2022). Dyslexia associated gene KIAA0319 regulates cell cycle during human neuroepithelial cell development. Front. Cell. Dev. Biol..

[28] Tarkar A., Loges N.T., Slagle C.E., Francis R., Dougherty G.W., Tamayo J.V., Shook B., Cantino M., Schwartz D., Jahnke C. (2013). DYX1C1 is required for axonemal dynein assembly and ciliary motility. Nat. Genet..

[29] Schueler M., Braun D.A., Chandrasekar G., Gee H.Y., Klasson T.D., Halbritter J., Bieder A., Porath J.D., Airik R., Zhou W. (2015). DCDC2 mutations cause a renal-hepatic ciliopathy by disrupting Wnt signaling. Am. J. Hum. Genet..

[30] Doust C., Fontanillas P., Eising E., Gordon S.D., Wang Z., Molz B., Pourcain B.S., Francks C., Marioni R.E., Zhao J. (2022). Discovery of 42 genome-wide significant loci associated with dyslexia. Nat. Genet..

[31] Gialluisi A., Andlauer T.F.M., Mirza-Schreiber N., Moll K., Becker J., Hoffmann P., Ludwig K.U., Czamara D., Pourcain B.S., Honbolygó F. (2021). Genome-wide association study reveals new insights into the heritability and genetic correlates of developmental dyslexia. Mol. Psychiatry.

[32] Lyytinen H., Erskine J., Hämäläinen J., Torppa M., Ronimus M. (2015). Dyslexia—Early Identification and Prevention: Highlights from the Jyväskylä Longitudinal Study of Dyslexia. Curr. Dev. Disord. Rep..

[33] Kovas Y., Plomin R. (2007). Learning Abilities and Disabilities: Generalist Genes, Specialist Environments. Curr. Dir. Psychol. Sci..

[34] Schmitz J., Abbondanza F., Paracchini S. (2021). Genome-wide association study and polygenic risk score analysis for hearing measures in children. Am. J. Med. Genet. B Neuropsychiatr. Genet..

[35] Brandler W.M., Paracchini S. (2014). The genetic relationship between handedness and neurodevelopmental disorders. Trends Mol. Med..

[36] Kere J. (2014). The molecular genetics and neurobiology of developmental dyslexia as model of a complex phenotype. Biochem. Biophys. Res. Commun..

[37] Zhao J., Yang Q., Cheng C., Wang Z. (2023). Cumulative genetic score of KIAA0319 affects reading ability in Chinese children: Moderation by parental education and mediation by rapid automatized naming. Behav. Brain Funct..

[38] Benchek P., Igo R.P., Voss-Hoynes H., Wren Y., Miller G., Truitt B., Zhang W., Osterman M., Freebairn L., Tag J. (2021). Association between genes regulating neural pathways for quantitative traits of speech and language disorders. NPJ Genom. Med..

[39] Chandrasekar G., Vesterlund L., Hultenby K., Tapia-Páez I., Kere J. (2013). The zebrafish orthologue of the dyslexia candidate gene DYX1C1 is essential for cilia growth and function. PLoS ONE.

[40] Anthoni H., Sucheston L.E., Lewis B.A., Tapia-Páez I., Fan X., Zucchelli M., Taipale M., Stein C.M., Hokkanen M.-E., Castrén E. (2012). The aromatase gene *CYP19A1*: Several genetic and functional lines of evidence supporting a role in reading, speech and language. Behav. Genet..

[41] Matsson H., Huss M., Persson H., Einarsdottir E., Tiraboschi E., Nopola-Hemmi J., Schumacher J., Neuhoff N., Warnke A., Lyytinen H. (2015). Polymorphisms in DCDC2 and S100B associate with developmental dyslexia. J. Hum. Genet..

[42] Luciano M., Gow A.J., Pattie A., Bates T.C., Deary I.J. (2018). The Influence of Dyslexia Candidate Genes on Reading Skill in Old Age. Behav. Genet..

[43] Matsson H., Tammimies K., Zucchelli M., Anthoni H., Onkamo P., Nopola-Hemmi J., Lyytinen H., Leppanen P.H.T., Neuhoff N., Warnke A. (2011). SNP variations in the 7q33 region containing DGKI are associated with dyslexia in the Finnish and German populations. Behav. Genet..

[44] Kong R., Shao S., Wang J., Zhang X., Guo S., Zou L., Zhong R., Lou J., Zhou J., Zhang J. (2016). Genetic variant in DIP2A gene is associated with developmental dyslexia in Chinese population. Am. J. Med. Genet. B Neuropsychiatr. Genet..

[45] Scerri T.S., Paracchini S., Morris A., MacPhie I.L., Talcott J., Stein J., Smith S.D., Pennington B.F., Olson R.K., DeFries J.C. (2010). Identification of candidate genes for dyslexia susceptibility on chromosome 18. PLoS ONE.

[46] Eicher J.D., Gruen J.R. (2015). Language impairment and dyslexia genes influence language skills in children with autism spectrum disorders. Autism Res..

[47] Mueller B., Ahnert P., Burkhardt J., Brauer J., Czepezauer I., Quente E., Boltze J., Wilcke A., Kirsten H. (2014). Genetic risk variants for dyslexia on chromosome 18 in a German cohort. Genes. Brain Behav..

[48] Yanpallewar S., Wang T., Koh D.C.I., Quarta E., Fulgenzi G., Tessarollo L. (2016). Nedd4-2 haploinsufficiency causes hyperactivity and increased sensitivity to inflammatory stimuli. Sci. Rep..

[49] WHO—World Health Organization (1992). International Statistical Classification of Diseases and Related Health Problems: 10th Revision (ICD-10).

[50] Kopanos C., Tsiolkas V., Kouris A., Chapple C.E., Aguilera M.A., Meyer R., Massouras A. (2019). VarSome: The human genomic variant search engine. Bioinformatics.

[51] Taipale M., Kaminen N., Nopola-Hemmi J., Haltia T., Myllyluoma B., Lyytinen H., Muller K., Kaaranen M., Lindsberg P.J., Hannula-Jouppi K. (2003). A candidate gene for developmental dyslexia encodes a nuclear tetratricopeptid.de repeat domain protein dynamically regulated in brain. Proc. Natl. Acad. Sci. USA.

[52] Ulfarsson M.O., Walters G.B., Gustafsson O., Steinberg S., Silva A., Doyle O.M., Brammer M., Gudbjartsson D.F., Arnarsdottir S., Jonsdottir G.A. (2017). 15q11.2 CNV affects cognitive, structural and functional correlates of dyslexia and dyscalculia. Transl. Psychiatry.

[53] Martinelli A., Rice M.L., Talcott J.B., Diaz R., Smith S., Raza M.H., Snowling M.J., Hulme C., Stein J., E Hayiou-Thomas M. (2021). A rare missense variant in the ATP2C2 gene is associated with language impairment and related measures. Hum. Mol. Genet..

[54] Grimm T., Garshasbi M., Puettmann L., Chen W., Ullmann R., Müller-Myhsok B., Klopocki E., Herbst L., Haug J., Jensen L.R. (2020). A novel locus and candidate gene for familial developmental dyslexia on chromosome 4q. Z. Für Kinder-Und Jugendpsychiatrie Und Psychother..

[55] Galesi O., Di Blasi F.D., Grillo L., Elia F., Giambirtone M.C., Figura M.G., Rizzo B., Buono S., Romano C. (2022). Dyslexia and Attention Deficit Hyperactivity Disorder Associated to a De Novo 1p34.3 microdeletion. Genes.

[56] Gerber P.J. (2011). The Impact of Learning Disabilities on Adulthood. J. Learn. Disabil..

[57] Visser M., Kayser M., Palstra R.J. (2012). HERC2 rs12913832 modulates human pigmentation by attenuating chromatin-loop for-mation between a long-range enhancer and the OCA2 promoter. Genome Res..

[58] Brancato D., Coniglio E., Bruno F., Agostini V., Saccone S., Federico C. (2023). Forensic DNA Phenotyping: Genes and Genetic Variants for Eye Color Prediction. Genes.

